# From Cytokine Storm to Cytokine Breeze: Did Lessons Learned from Immunopathogenesis Improve Immunomodulatory Treatment of Moderate-to-Severe COVID-19?

**DOI:** 10.3390/biomedicines10102620

**Published:** 2022-10-18

**Authors:** Goran Rondovic, Dragan Djordjevic, Ivo Udovicic, Ivan Stanojevic, Snjezana Zeba, Tanja Abazovic, Danilo Vojvodic, Dzihan Abazovic, Wasim Khan, Maja Surbatovic

**Affiliations:** 1 Clinic of Anesthesiology and Intensive Therapy, Military Medical Academy, Crnotravska 17, 11000 Belgrade, Serbia; 2 Faculty of Medicine of the Military Medical Academy, University of Defence, Crnotravska 17, 11000 Belgrade, Serbia; 3 Institute for Medical Research, Military Medical Academy, Crnotravska 17, 11000 Belgrade, Serbia; 4 Biocell Hospital, Omladinskih Brigada 86a, 11000 Belgrade, Serbia; 5 Division of Trauma & Orthopaedic Surgery, University of Cambridge, Addenbrooke’s Hospital, Cambridge CB2 2QQ, UK

**Keywords:** COVID-19, moderate-to-severe pneumonia, cytokines, immunologic, immunosuppression, immunotherapy, anakinra, critical illness

## Abstract

Complex immune response to infection has been highlighted, more than ever, during the COVID-19 pandemic. This review explores the immunomodulatory treatment of moderate-to-severe forms of this viral sepsis in the context of specific immunopathogenesis. Our objective is to analyze in detail the existing strategies for the use of immunomodulators in COVID-19. Immunomodulating therapy is very challenging; there are still underpowered or, in other ways, insufficient studies with inconclusive or conflicting results regarding a rationale for adding a second immunomodulatory drug to dexamethasone. Bearing in mind that a “cytokine storm” is not present in the majority of COVID-19 patients, it is to be expected that the path to the adequate choice of a second immunomodulatory drug is paved with uncertainty. Anakinra, a recombinant human IL-1 receptor antagonist, is a good choice in this setting. Yet, the latest update of the COVID-19 Treatment Guidelines Panel (31 May 2022) claims that there is insufficient evidence to recommend either for or against the use of anakinra for the treatment of COVID-19. EMA’s human medicines committee recommended extending the indication of anakinra to include treatment of COVID-19 in adult patients only recently (17 December 2021). It is obvious that this is still a work in progress, with few ongoing clinical trials. With over 6 million deaths from COVID-19, this is the right time to speed up this process. Our conclusion is that, during the course of COVID-19, the immune response is changing from the early phase to the late phase in individual patients, so immunomodulating therapy should be guided by individual responses at different time points.

## 1. Introduction

Since the beginning of the COVID-19 pandemic, complex immune response to the SARS-CoV-2 virus has been the focus of both clinicians and researchers. Very early on, in April 2020, an important hypothesis-generating paper regarding SARS-CoV-2 and viral sepsis was published [1], emphasizing that a process called viral sepsis is crucial to the disease mechanism of COVID-19. The authors also stated that the efficacy of immunomodulatory therapies should be assessed in RCTs. Due to an inadequate host response to the initial viral replication phase, COVID-19 is, by definition, viral sepsis [2]. Complex immune response as well as generalized thrombotic endotheliopathy might be common features of bacterial and viral sepsis. Therefore, antiviral drugs have a limited effect in severe forms of COVID-19. Bearing in mind that sepsis is a highly heterogenous syndrome, it should be noted that there are different phenotypes in critically ill COVID-19 patient populations that fulfill SEPSIS-3 criteria in most cases. Multiple organ dysfunction syndrome (MODS) accounts for COVID-19 mortality rates, which are comparable to those of other viral, bacterial or fungal sepsis.

Nevertheless, COVID-19 patients are more homogeneous than the general sepsis patient population [3]. It is, therefore, of paramount importance to better select patients who would benefit the most from a particular treatment both for COVID-19 and other sepsis patient subgroups. One of the first comprehensive reviews regarding the immunopathogenesis, transmission, diagnosis and treatment of COVID-19 available at the time was published in mid-2020 [4]. With time, it became clearer that there are some differences in immune response between bacterial sepsis and COVID-19. The RCI-COVID-19 study group demonstrated that immune suppression is much more pronounced in bacterial septic shock patients compared with severe COVID-19 patients based on monocytic human leukocyte antigen–DR isotype (HLA-DR) expression, which is significantly lower in bacterial septic shock patients (*p* < 0.0001) [5]. In addition, the so-called “cytokine storm” may be nothing more than a “tempest in a teapot” in severe COVID-19, with much lower cytokine levels compared with critically ill non-COVID-19 patients [6]. A “cytokine storm” is defined by overwhelming hyperinflammation due to a surge of inflammatory molecules [7,8,9,10,11,12,13,14,15], while “cytokine breeze” is consistent with modestly elevated levels of inflammatory mediators [16]. This was confirmed in other studies and will be discussed in more detail in this review. The authors of an interesting study, regarding the immunopathophysiology of COVID-19 as community-acquired pneumonia (CAP) caused by SARS-CoV-2, demonstrated the diverging immune features of COVID-19 and influenza using integrated single-cell analysis of peripheral immune cells [17]. Perhaps the most striking difference between severe COVID-19 and bacterial sepsis, apart from cytokine levels, is immunothrombosis. There are several terms in use in clinical and research settings, COVID-19-associated coagulopathy (CAC), sepsis-induced coagulopathy (SIC), pulmonary intravascular coagulopathy (PIC), in contradistinction to disseminated intravascular coagulopathy (DIC). All these processes are the focus of intense investigation [18,19]. In COVID-19 patients, the simultaneous activation of coagulation and inhibition of fibrinolysis has been demonstrated with increased levels of plasminogen activator inhibitor 1 (PAI-1) and decreased levels of d-dimer in comparison with sepsis patients [20].

Finally, in a recently published thorough overview of the evidence regarding immunotherapy for COVID-19, it was emphasized that challenges in clinical decision-making arise from the complexity of the disease phenotypes and patient heterogeneity, as well as the variable quality of evidence from immunotherapy studies [21]. The effects of anti-IL-1 therapy (anakinra) might be a good example of the need for a more personalized approach to identify who will benefit the most. The CORIMUNO-ANA-1 randomized controlled trial regarding the effect of anakinra versus usual care in adults in the hospital with COVID-19 and mild-to-moderate pneumonia was a negative one [22]. The authors concluded that anakinra did not improve outcomes in patients with mild-to-moderate COVID-19 pneumonia. However, a biomarker (soluble urokinase plasminogen activator receptor—suPAR)-guided approach to the same issue proved important for patients because the results of a double-blind, randomized control phase 3 trial of anakinra in the early treatment of severe COVID-19 patients with elevated suPAR levels (SAVE-MORE) were positive [23]. The authors demonstrated that in the anakinra arm, 28-day mortality was lower (3.9% vs. 8.7% in controls).

Precision medicine for COVID-19 was the focus of a good review in which the authors discussed how not all SARS-CoV-2 hosts and host responses are the same [24]. COVID-19 is still a multifaceted puzzle that should be solved [25].

## 2. Aspects of Host Response—How Specific Are They for COVID-19?

**Figure 1:**
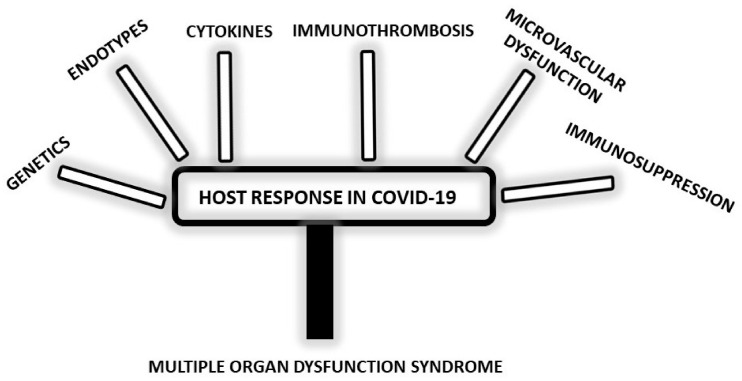
Key elements of host response in COVID-19.

This section of the review is dedicated to the complexity of the host response to the pathogen in COVID-19. Key elements are summarized in Figure 1.

### 2.1. Genetic Point of View

Bacterial sepsis and severe COVID-19 have been investigated from a genetic point of view, focusing on potential differences in gene signature. Myeloid-derived suppressor cells (MDSCs) have been extensively investigated in bacterial sepsis by many investigators, including our group [26,27]. An in-depth analysis of these fascinating cells was performed by Reyes et al. using single-cell RNA-sequencing to profile the blood of patients with bacterial sepsis [28]. They demonstrated an expanded CD14+ monocyte state called MS1, which is reminiscent of MDSCs. The same group showed that the expression of the MS1 and class II major histocompatibility complex (MHC-II) gene program is associated with sepsis severity and outcomes in patients with bacterial sepsis or COVID-19. Plasma from patients with bacterial sepsis or severe COVID-19 induces suppressive myeloid cell production from hematopoietic progenitors in vitro [29]. The characterization of the inflammatory signatures of peripheral blood mononuclear cells from patients with COVID-19, bacterial sepsis and HIV infection was performed by integrative analysis of single-cell transcriptomes in [30]. The authors identified 10 hyperinflammatory cell subtypes with monocytes as the main contributors to the transcriptional and immune response differences, while T cells had a common role in host responses to all of these infections. Furthermore, in COVID-19, there are some common hyperinflammatory genes with sepsis, as well as some common immunosuppressive genes with HIV infection. However, COVID-19 has a very specific immune cell signature. Mono-CD14-IFITM3 and Mono-CD14-CD16 are two monocyte subtypes responsible for initiating the interferon signaling pathway in response to infection and are specific to COVID-19 patients. Overall, genes in COVID-19 and sepsis are differentially expressed. In this study, heterogeneity among COVID-19 patients was analyzed, and a “three-stage” model was identified that related to the hyperinflammatory and immunosuppressive signatures in monocytes. Three COVID-19 clusters (from moderate to severe forms) were identified, which may provide therapeutic guidance to improve treatments for subsets of COVID-19 patients. In recently published research, Carapito et al. used multi-omics analyses combined with artificial intelligence to identify the specific driver genes of critical COVID-19 in a young, comorbidity-free patient cohort [31]. The authors demonstrated a specific peripheral blood mononuclear cell (PBMC) pattern in critical COVID-19 using an immune profiling assay of PBMCs. Memory CD4 and CD8 T cells, as well as Th17 cells, decreased with disease severity. Memory B cells also decreased, while naïve B cells and plasmablasts increased; non-classical monocytes decreased, presumably due to migration to the lung. Dendritic cells decreased with disease severity. PBMC proteomics was also performed and showed dysregulated coagulation proteins, as well as macrophage and myeloid cell compartments. The robust gene expression signature in this investigation differentiated critical and noncritical COVID-19 patients, with five genes of particular interest in this regard. One of them, a disintegrin and a metalloprotease 9 (ADAM9) gene, was significantly upregulated in critically ill COVID-19 patients. The ex vivo silencing of ADAM9 is associated with less SARS-CoV-2 uptake by the cells as well as less viral replication. Thus, there are some similarities between COVID-19 and other infectious diseases. Nevertheless, COVID-19 has a specific immune cell signature, while critical COVID-19 has a specific gene signature.

### 2.2. Importance of Different Sepsis Endotypes

For a better understanding of sepsis endotypes, research from the field of rheumatology field is relevant. For the classification of sepsis patients, among other elements, a hemophagocytosis score (HS) can be helpful. In the pre-COVID era, a minority of patients with sepsis, around 4%, scored positive and experienced secondary macrophage activation-like syndrome (MALS), an independent, life-threatening entity in sepsis. Ferritin measurements can provide an early diagnosis of MALS and may allow for specific treatment [32]. Early in the COVID-19 pandemic, in mid-2020, Bourboulis et al. published an excellent study on complex immune dysregulation in COVID-19 patients [33]. Patients with pneumonia but without progression to acute respiratory distress syndrome (ARDS) maintained HLA-DR expression in monocytes with vivid antigen presentation, a moderate elevation of C-reactive protein (CRP), d-dimer and alanine transaminase (ALT)/aspartate transaminase (AST). A quarter of the patients with progression to COVID-19-associated ARDS presented with macrophage activation syndrome: high levels of CRP, ferritin, d-dimer, AST/ALT, tumor necrosis factor (TNF)-alpha, interleukin (IL)-1beta and IL-6. In total, 25% of severe COVID-19 patients had moderate antigen presentation. Three-quarters of these patients presented with immune dysregulation: very low HLA-DR expression, profound depletion of lymphocytes and natural killer (NK) cells, as well as weak antigen presentation. This resembles sepsis-induced immunosuppression. However, there was a pattern in 75% of patients that was distinct from bacterial sepsis: TNF-alpha and IL-6 production by circulating monocytes were sustained; furthermore, there were high levels of CRP, d-dimer and AST/ALT. The authors used the term complex immune dysregulation for this unexpected severe COVID-19 endotype. An unsupervised analysis of transcriptomics in bacterial sepsis was performed by Sweeney et al. This group, using a unified clustering analysis across 14 discovery datasets with 600 patients, revealed three subtypes, inflammopathic, adaptive and coagulopathic, based on functional analysis. The authors built the classifier, a total of 33 genes, which assigns each sample three scores (one for each cluster type) and then applies multiclass regression to output a final cluster assignment [34]. This approach of inflammopathic, adaptive, and coagulopathic sepsis endotypes was validated in COVID-19 using predefined a 33-messenger RNA endotype classifier [35]. In this study, the endotype was the moderator of prognosis. Of 97 COVID-19 patients, 29% were inflammopathic, 44% were adaptive and 27% were coagulopathic. An association with the outcome in all three endotypes points to the heterogeneity of severe COVID-19. In the inflammopathic endotype, there were five genes of interest, mortality was 18 % and patients had higher levels of CRP and IL-6 resembling macrophage activation. In the adaptive endotype, there were 17 genes of interest, the patients had lower rates of ARDS and no deaths were seen resembling T cell activation. Finally, in the coagulopathic endotype, there were 11 genes of interest, patients had the highest mortality rate of 42% and high levels of d-dimer were seen resembling endothelial activation. Based on the current literature, there are some differences between COVID and non-COVID sepsis regarding endotypes. In non-COVID sepsis, there is great heterogeneity, with about 5% of patients in pure hyperinflammation, about 25% of patients with pure immunosuppression and the majority (around 70%) in between these two extremes, presenting mixed findings. COVID sepsis is more homogenous, with 25% of patients in pure hyperinflammation and most of them in complex dysregulation (which is not real immunoparalysis). Endotypes can change over time, and this kinetic feature has to be taken into account in both COVID and non-COVID sepsis. This is the field where theragnostics can guide the future.

### 2.3. Complexity of Cytokine Response

A simplified scheme of the severe form of COVID-19 encompasses cytokine-driven hyperinflammation, lymphopenia, dysregulated inflammatory response in the systemic circulation, myeloid hyperinflammation and dysfunctional type I interferon (IFN) response. Given that SARS-CoV-2 internalizes via angiotensin-converting enzyme 2 (ACE2) within the host, the resultant epithelial disruption in ACE2 expression can alter tissue function and contribute to ARDS development because of the abundant presence of ACE2 in respiratory epithelia [36]. The catchy term “cytokine storm” stuck early on during the pandemic, both among the general public and in the scientific literature. Experts, however, challenged this undefined term, suggesting that it should be synonymous with COVID-19 pathophysiology [6]. One study showed that levels of TNF-α, IL-6 and IL-8 were significantly lower in COVID-19 patients than in patients with bacterial sepsis with or without ARDS [37]. Investigators demonstrated that levels of these three cytokines were similar to those seen in critically ill patients with no infection, such as in out-of-hospital cardiac arrest (OHCA) or trauma. The results of a rapid systematic review, meta-analysis and comparison of cytokine elevation in severe and critical COVID-19 with other inflammatory syndromes in [38] were in line with the notion of COVID-19 not being a “cytokine storm” at all. In that paper, regarding IL-6 levels in COVID-19 versus other disorders, the authors demonstrated that the estimated pooled mean for IL-6 concentrations in all COVID-19 patients, both severe and critical, was only 36.7 pg/mL. In all other disorders, pooled mean IL-6 concentrations were significantly higher: in patients with hypoinflammatory ARDS, 198.6 pg/mL (5-fold higher); in those with hyperinflammatory ARDS, 1558.2 pg/mL (40-fold higher); in all ARDS patients, 460.1 pg/mL (12-fold higher). In more than 5000 patients with non-COVID sepsis, the pooled mean IL-6 level was 983.6 pg/mL. The so-called “cytokine storm” was present only in patients with chimeric antigen receptor (CAR) T cell-induced cytokine release syndrome—CRS—and the pooled mean IL-6 serum concentration was 3110.5 pg/mL, nearly 100-fold higher than in the severe and critical COVID-19 patient populations. The authors noted that 80% of COVID-19 studies report mean IL-6 concentrations lower than 100 pg/mL. A similar pattern was found for IL-8 concentrations in these eight categories of patients; the mean IL-8 concentration was 22 pg/mL in patients with COVID-19, while it was 228 pg/mL (10-fold higher) in patients with sepsis. In patients with CRS, the mean concentration of IL-8 was 575 pg/mL. The mean concentration of proximal proinflammatory mediator TNF-alpha was 5 pg/mL in COVID-19 patients, 34.6 pg/mL in patients with sepsis and 52.2 pg/mL in patients with CRS. More than 90% of COVID-19 studies (all but one) reported mean a TNF-alpha concentration lower than 10 pg/mL. Thus, there is an obvious pattern in concentrations of proinflammatory cytokines. Moreover, the potent anti-inflammatory cytokine IL-10 followed the same pattern: mean concentrations in severe and critical COVID-19 patients were the lowest, mostly below 50 pg/mL; in patients with sepsis, the mean IL-10 concentration was just below 400 pg/mL; and in CRS patients, it was highest at nearly 800 pg/mL. Although the high inter- and intraindividual variabilities in cytokine levels are well known, the pattern seen in this review for the various cytokines was consistent between different patient populations. The authors of this seminal article proposed a very interesting mechanistic comparison of inflammatory processes in patients with COVID-19 versus ARDS, sepsis and CAR T cell-induced CRS in all three important compartments (cellular, interstitial and blood), including not only cytokines but also acute phase reactants (d-dimer, CRP, ferritin, lactate dehydrogenase—LDH), as well as lymphocytes and neutrophiles. Schuurman et al. performed integrated single-cell transcriptomic and proteomic analyses in peripheral blood mononuclear cells from a matched cohort of eight patients with COVID-19, eight with community-acquired pneumonia caused by Influenza A or other pathogens, and four non-infectious control subjects [17]. The authors described both shared and diverging transcriptional and phenotypic patterns, demonstrating that T cells, as well as NK cells from COVID-19 patients, exhibit a clear type I interferon signature and underscoring the peripheral immune response in different etiologies of pneumonia. All data regarding both systemic and compartmentalized immune responses are of immense clinical importance. For example, it was established a few years ago that plasma cytokine levels predict responses to corticosteroids in septic shock patients [39]. The authors of the study demonstrated that the beneficial effect of corticosteroid treatment on 28-day survival in propensity-matched patients depends on the cytokine level threshold. They investigated over a dozen cytokines, including IL-6 and IL-4, in patients with septic shock. It was revealed that patients below the cytokine level threshold with no corticosteroid treatment had the best survival, followed by patients above the threshold with corticosteroid treatment. On the other hand, patients with cytokine levels below the threshold who were corticosteroid-treated had worse outcomes. The highest mortality was in patients with cytokine levels above the threshold and without corticosteroid treatment.

### 2.4. Immunothrombosis as Coagulopathy

The coagulation system evolved as an effector pathway of the immune response, laying down fibrin around pathogens to physically entrap them and prevent their dissemination. Thus, the endpoint of inflammation is thrombosis. Immunothrombosis is one of the key features of both COVID-19 and non-COVID-19 sepsis. Whatever the underlying disorder is, systemic disseminated intravascular coagulation (DIC) will encompass widespread fibrin deposition, leading to microvascular thrombotic obstruction and ultimately organ failure, as well as the consumption of platelets and clotting factors, leading to thrombocytopenia and coagulation factor deficiency, with both bleeding and thrombosis as a consequence of this derangement. In sepsis, monocytes activated by proinflammatory cytokines (TNF-alpha, IL-1beta) will express tissue factor (TF) on their surface. TF will initiate local coagulation and is, therefore, the key to septic DIC. In COVID-19 pathogenesis, the prothrombotic state is due to the effects of IL-1 and IL-6. Early in the pandemic, Desborough et al. investigated coagulation and inflammation markers as part of an acute phase response in 66 patients with severe COVID-19 [40]. Clauss fibrinogen, d-dimer, procalcitonin (PCT), ferritin and CRP were compared between mechanically ventilated patients with and without proven venous thromboembolism (VTE). Both clauss fibrinogen and d-dimer were extremely high in this group of patients but did not discriminate between those who had a proven clot. There was no statistically significant difference between VTE and no-VTE patients in terms of ferritin, PCT or CRP levels. SARS-CoV-2 causes endothelial cell activation and the switching of the anti-coagulant phenotype (the production of nitric oxide—NO—to downregulate platelets and activate antithrombin, thrombomodulin and protein C) to a pro-coagulant one, producing potent phospholipid mediator platelet-activating factor (PAF). COVID-19 is a perfect recipe for VTE in critically ill immobile patients, with profound prothrombotic changes in the endothelial lining of the vessel wall and within the blood. Therefore, all elements of the Virchow triad of thrombosis are present: blood stasis, hypercoagulability and vessel wall injury. This may lead to deep vein thrombosis and pulmonary embolisms. On the other hand, local inflammation and immunothrombosis within the lung microvasculature, as in COVID-19 ARDS, may lead to pulmonary thrombosis. Immunothrombosis is a reminder that coagulation is the endpoint or effector system of immune responses. Many prothrombotic mechanisms occur in patients with COVID-19 pneumonia. For instance, high activation of neutrophils causes NETOSIS (formation of neutrophil extracellular traps (NETs), which consist of modified chromatin). Platelet activation is also present, including microparticle production; there is heightened platelet–neutrophil interaction, resulting in crosstalk between immunity and coagulation. The net effect of cytokines is downregulating fibrinolysis in DIC. Fibrinolytic shutdown is due to increased plasminogen activator inhibitor-1 (PAI-1), also known as endothelial plasminogen activator inhibitor or serpin E1. PAI-1 is a serine protease inhibitor (serpin) that functions as the principal inhibitor of the tissue plasminogen activator (tPA) and urokinase-type plasminogen activator (uPA), the activators of plasminogen and, hence, fibrinolysis. There is a paradox in severe COVID-19: fibrinolytic shutdown but very increased d-dimers (the breakdown product of fibrin degradation) [41]. The hallmark of acute lung injury is intra-alveolar fibrin deposition, and later remodeling of fibrin may lead to lung fibrosis. Hunt and Levi proposed an alternative hypothesis regarding increased levels of d-dimer in this setting [42]. They argued that d-dimers originate from damaged lung tissue because pneumocytes can locally produce uPA to break down local clots due to immunothrombosis. Extravascular proteolysis, inhibited by PAI-1, is regulated in the lung tissue. Another hallmark of COVID-19 pneumonia lung histology is an abundance of activated lung macrophages, which can generate plasmin and matrix metalloproteinases (MMPs) for the proteolysis of fibrin monomers. These cells can grab passing fibrin molecules, pull them in and endocytose them, break them down and discard the debris. In their opinion, d-dimers are such good predictors of outcomes because they reflect the extent of lung injury, and COVID-19 produces the most prothrombotic state ever seen with a viral infection. In the kidney and many other cells, uPA is released as a single-chained molecule and converted to two-chained uPA by plasmin. After that, uPA binds to its receptor—uPAR—anchored to the cell surface. This anchor can be proteolyzed, and uPAR will be free from the cell surface and can be detected in plasma as soluble uPAR, that is, suPAR. High plasma levels of suPAR in severe COVID-19 predict the extent of organ injury [43]. The pathogenesis of COVID-19 coagulopathy is a mix of acute phase response, endothelial cell activation, fibrinolytic shutdown and increased levels of d-dimers due to overspill from the lungs [42]. It is not DIC, because fibrinogen levels are high (low in DIC); activated partial thromboplastin clotting time (aPTT)/prothrombin time (PT) and platelet count tend to be normal in COVID-19 coagulopathy. In DIC, aPTT/PT is prolonged and platelet count is low. However, critically ill COVID-19 patients dying of hypoxia can develop DIC because hypoxia, by itself, is a big driver of DIC. COVID-19 and sepsis are associated with different abnormalities in plasma procoagulant and fibrinolytic activity [44]. Investigators compared the biomarkers of endogenous coagulation and fibrinolytic activity, thrombin and plasmin generation potential and fibrin formation and lysis between COVID-19 patients, sepsis patients and healthy donors. Although d-dimer came into focus with COVID-19, it was demonstrated that levels of d-dimer were, statistically, significantly higher in sepsis than in COVID-19. An interesting comparison was made of coagulopathies associated with COVID-19 and sepsis encompassed levels of d-dimer, circulating extracellular vesicle tissue factor (EVTF) activity and active PAI-1 in plasma samples [20]. The authors demonstrated that levels of d-dimer, which is a marker of fibrin dissolution, were significantly lower in patients with COVID-19 compared with sepsis, while the opposite was true for active PAI-1, with levels significantly higher in patients with COVID-19 compared with sepsis. They concluded that patients with COVID-19 have higher levels of an activator of coagulation EVTF, as well as an inhibitor of fibrinolysis, PAI-1, compared with sepsis patients. COVID-19 thus promotes thrombosis rather than DIC, which is the opposite of sepsis, where DIC is favored over thrombosis. The pulmonary procoagulant response in COVID-19 patients with persistent ARDS was investigated by Nossent et al. [45]. The authors concluded that results suggest a local pulmonary rather than a systemic procoagulant and inflammatory “storm” in severe COVID-19. In line with the above-mentioned study was a review focused on pulmonary intravascular coagulopathy (PIC) [46]. Immunothrombosis in COVID-19 appears to be more extensive than in other viral pneumonias. The mechanism of immunothrombosis is very important because it explains why anticoagulants do not improve outcomes in these states. Therefore, instead of this therapeutic approach, the inflammatory process should be treated, bearing in mind that innate inflammatory response is initiated by pathogen-associated molecular patterns (PAMPs) and endogenous damage-associated molecular patterns (DAMPs) produced by damaged cells. DAMPs are released in sepsis, trauma and various other states [47].

### 2.5. Endothelial and Microvascular Dysfunction

COVID-19-induced inflammation and endothelial activation and/or dysfunction can promote microvascular dysfunction. Sublingual microcirculation can be assessed using handheld vital microscopes. In the MYSTIC study, COVID-19 patients had an up to 90% reduction in vascular density [48]. Increased levels of angiopoietin-2, tyrosine-protein kinase receptor (Tie-2), syndecan-1, hyaluronic acid, thrombomodulin and ACE2 indicate endothelial injury, while microcirculation derangement includes decreased RBC velocity, capillary density and glycocalyx thickness. Both are present in COVID-19 patients, intertwined with immune dissonance. COVID-19 sublingual microcirculatory alterations caused by inflammation, coagulopathy and hypoxemia were investigated by Favaron et al. [49]. COVID-19 patients had increased total vessel density, functional capillary density, proportions of the perfused vessel, RBC velocity, capillary hematocrit, and capillary-hematocrit-to-systemic-hematocrit ratio in comparison with healthy controls. All these effects were demonstrated in COVID-19 patients with less severe forms of the disease, i.e., with Sequential Organ Failure Assessment (SOFA) scores less than 10, but not in patients with SOFA scores of 10 or more. This interesting study suggests that patients are able to adapt to hypoxia and hypoxemia to a certain extent, but this adaptation is not possible when inflammation and endothelial dysfunction are more pronounced. In another interesting study, investigators focused on the microcirculatory, endothelial and inflammatory responses in critically ill COVID-19 patients in comparison with a group of patients with septic shock using a prospective observational case–control study [50]. They performed measurements of sublingual microcirculatory flow with video-microscopy and serial measurements of IL-6 and syndecan-1 levels. Septic patients had significantly worse microcirculatory flow and higher IL-6 levels than those with COVID-19, while syndecan-1 levels were similar. The authors concluded that there was no evidence of significant sublingual microcirculatory impairment, widespread endothelial injury or marked inflammatory cytokine release in this group of critically ill COVID-19 patients. The aforementioned data suggest that microvascular dysfunction in COVID-19 is determined by macrocirculation, hypoxemia, inflammation, endothelial injury and hypercoagulopathy. The ability of the microcirculation to adapt to hypoxemia may be impaired as the endothelium and glycocalyx are more damaged and a procoagulant state develops. Microvascular and endothelial dysfunction are hence correlated with disease severity in COVID-19.

### 2.6. Immunosuppression in COVID-19

Immunosuppression can be present in COVID-19 patients, and this demonstrates the importance of immune endotype determination. If profound immunosuppression predominates, the therapeutic approach is diametrically different compared with COVID-19 patients with hyperinflammation. Early on, in 2020, it was pointed out that the “cytokine storm” might be episodic while lymphopenia is sustained in critically ill COVID-19 patients, which correlates with increased secondary infections and death [51]. Furthermore, in this setting, T cell depletion and exhaustion induce immunosuppression, which contributes to incessant viral load and a lethal outcome [52]. Invasion by the virus can also be facilitated by an inadequate humoral immune response. If antibodies produced by immune cells are not able to neutralize the virus, antibody-dependent enhancement (ADE) might occur. In this setting, virus–antibody complexes can bind to Fc or other receptors on host cells, thereby facilitating virus invasion [53]. Reduced mHLA-DR expression indicates immunosuppression in critically ill COVID-19 patients [54]. A very interesting study highlighted immunosuppression as a hallmark of COVID-19 evolution [55]. Monocytes from COVID-19 patients who had a fatal outcome in ICU were dysfunctional and had lost immune regulatory properties. This finding is comparable to immune paralysis, when monocytes in septic patients do not respond to lipopolysaccharide (LPS) stimulation with the upregulation of nuclear factor (NF)-κB-dependent genes, including TNF. The authors suggested a state of “immune silence” that correlates with severe clinical manifestation and fatal outcomes, which could be related to the extensive immaturity of the cell population because of an abnormal and skewed myelopoiesis, together with a reset of the lymphoid arm indicated by the accumulation of naïve T cells. They also suggested administering drugs that can “reawaken” the host immune system.

### 2.7. COVID-19 Post-Mortem Point of View—Evidence of MODS

One meta-analysis demonstrated that almost 80% of hospitalized COVID-19 patients fulfill the SEPSIS-3 definition using the SOFA score [56]. This is important for many reasons, including the fact that COVID-19 patients die primarily from multiple organ dysfunction syndrome. The systemic nature of this disease is confirmed in post-mortem studies; one cohort autopsy study demonstrated permanent viral presence in various tissues [57]. Another interesting study focused on causes of death and comorbidities in hospitalized patients with COVID-19 [58]. Full autopsies of 26 COVID-19 patients demonstrated that septic shock and MODS were the most common immediate causes of death, often due to suppurative pulmonary infection. Respiratory failure due to diffuse alveolar damage (DAD) presented as the immediate cause of death in fewer cases. Death may be caused by the thrombosis observed in segmental and subsegmental pulmonary arterial vessels [59]. The high incidence of thromboembolic events suggests the important role of COVID-19-induced coagulopathy. Autopsies in one study revealed deep venous thrombosis in 7 of 12 patients (58%), in whom venous thromboembolism was not suspected before death; pulmonary embolism was the direct cause of death in 4 of 12 patients [60]. Liver involvement in COVID-19 was also confirmed in post-mortem studies. Large amounts of platelet–fibrin microthrombi in the hepatic sinusoids, central vein or portal vein in COVID-19 livers, as well as steatosis, and lobular and portal tract inflammation was demonstrated in one study [61]. Yet, the authors of another post-mortem study demonstrated that the main histological changes can be explained by the hypoxic status as a result of severe hypoxemic pneumonia leading to death. Drug toxicity may also play a role in certain cases. They concluded that COVID-19 infection was not associated with a specific histopathological pattern of the liver [62]. In any case, one recently published meta-analysis demonstrated that the severity of COVID-19 was associated with aberrant liver function tests [63]. Interesting findings regarding the temporal evolution of COVID-19-associated cardiopathy were reported by Haslbauer et al. [64]. The investigators found early microvascular dysfunction, which was followed by secondary cardiac inflammatory infiltrates with or without necrosis in other groups of RT-PCR-negative cases, along with a longer hospitalization time. Gross cardiac pathology findings from another post-mortem study [65] revealed left ventricular dilation in 52% of the COVID-19 cases and right ventricular dilation in 56%, while microscopic findings revealed cardiac microthrombi in 66% of the cases. Another pathologic study confirmed that microthrombi are a major cause of cardiac injury in COVID-19 [66]. Among a cohort of 42 patients dying of COVID-19, autopsy histologic kidney evaluations revealed acute tubular injuries, which were typically mild relative to the degree of creatinine elevation. These findings suggest the potential for recovery upon resolution of SARS-CoV-2 infection. The authors also reported the absence of classic viral nephropathy or diffuse thrombotic microangiopathy [67]. An interesting COVID-19-association-dependent categorization (“strong”, “contributive” or “weak”) of death causes in 100 autopsy cases was performed by Danics et al. [68].

## 3. Immunomodulatory Treatment of Moderate-to-Severe COVID-19—Did We Learn the Lesson?

The life-threatening COVID-19 pandemic created a unique situation with a large number of drugs being evaluated in a very short period of time. The use of hydroxychloroquine in intensive care unit (ICU) patients is an example of wasted resources, where these critically ill COVID-19 patients could have been included in other trials. In an interesting study regarding experimental and compassionate drug use during the first wave of the COVID-19 pandemic [69], the authors highlighted that this kind of treatment was unrelated to survival. A good conceptual model of the time-course of COVID-19 infection from the asymptomatic phase to more severe phases in patients who develop critical illness was proposed by Angriman et al. [70]. The authors divided the clinical stage of the disease into five phases: asymptomatic phase, mild illness, moderate-to-severe disease (need for oxygen support but not in the ICU), critical disease and prolonged critical illness (both in the ICU). They also highlighted the main physiological features of each phase. In the asymptomatic phase and mild disease, high functional reserve and viral replication are dominant. In moderate-to-severe disease, inflammatory response, the secondary infection risk and mortality risk rise, viral replication is very low and there is still functional reserve present. In critical disease, there is a peak in the inflammatory response, and secondary infection risk and mortality risk are high while functional reserve declines rapidly. In prolonged critical illness, there is the highest level of secondary infection risk and mortality risk as expected, with very low functional reserve. The fact that the intensity of the immune response is rather low in this phase is crucial for immunomodulatory treatment because the time window for intervention is most likely closed, and immune modulation is potentially harmful at this end stage. Current therapies under investigation for COVID-19 in phase III can be divided into early interventions and treatments for inflammatory response. In the early intervention group, apart from monoclonal antibodies and antivirals, are corticosteroids, including dexamethasone and inhaled budesonide. Treatments for the inflammatory response phase encompass several subgroups of compounds with different mechanisms of action. For IFNbeta-1a, the rationale relies on evidence of anti-viral activity against SARS-CoV-2 in vitro and in animal models. Anti-cytokines (anti-IL-6/IL-1) include sarilumab, tocilizumab, canakinumab and anakinra. The monoclonal antibody vilobelimab (IFX-1—a chimeric monoclonal IgG4 antibody) selectively blocks potent anaphylatoxin C5a. The next group consists of otilimab, a fully human monoclonal antibody that inhibits granulocyte–macrophage colony-stimulating factor (GM-CSF) and mavrilimumab, a human monoclonal antibody that inhibits human granulocyte–macrophage colony-stimulating factor receptor (GM-CSF-R). Finally, there is the Janus kinase (JAK) group inhibitors, including baricitinib and tofacitinib; fostamatinib is a spleen tyrosine kinase inhibitor and imatinib is a specific tyrosine kinase receptor inhibitor.

**Figure 2:**
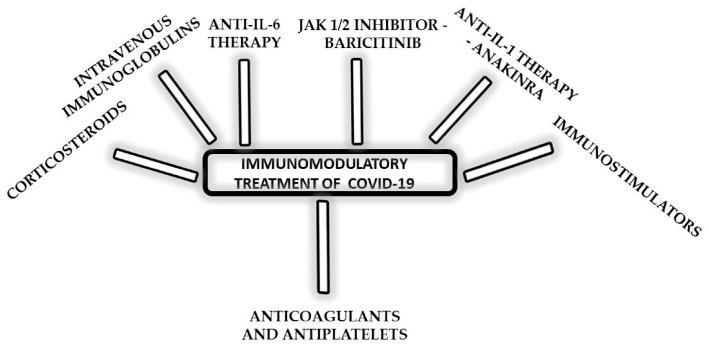
Key elements of the immunomodulatory treatment of COVID-19.

COVID-19 treatment guidelines are constantly evolving, with many versions of protocols. Current US NIH guidelines for hospitalized patients who do not require supplemental oxygen recommend against the use of dexamethasone or other corticosteroids. For this group of patients, without evidence of VTE, a prophylactic dose of heparin is recommended. For hospitalized patients who require supplemental oxygen, dexamethasone and a prophylactic dose of heparin are recommended. For patients on dexamethasone with rapidly increasing oxygen needs and systemic inflammation, either baricitinib or tocilizumab should be added as a second immunomodulatory drug. The same goes for patients who require supplemental oxygen through the high-flow device of noninvasive ventilation. Finally, for patients who require mechanical ventilation (MV) or extracorporeal membrane oxygenation (ECMO), dexamethasone and tocilizumab (alternatively, sarilumab) within 24 h of ICU admission are recommended [71]. The key elements of the immunomodulatory treatment of COVID-19 are summarized in Figure 2.

### 3.1. Corticosteroids for All COVID-19 ICU Patients?

It is obvious that corticosteroids are considered the backbone of immunomodulatory treatment. However, not all questions regarding this potent therapy have yet been answered. One question is whether corticosteroids should be used to treat all ICU patients with COVID-19 pneumonia. Surviving Sepsis Campaign (SSC) guidelines on the management of critically ill COVID-19 patients were originally made available on 28 March 2020 [72]. In this version, it was suggested that systemic corticosteroids should not routinely be used in mechanically ventilated patients with COVID-19 respiratory failure (without ARDS). Like most other recommendations, this did not have a strong evidentiary basis. This was followed by a controlled, open-label trial where hospitalized COVID-19 patients were randomized either to receive dexamethasone (at a dose of 6 mg once daily) for up to 10 days or to receive standard of care (SOC). The use of dexamethasone resulted in lower 28-day mortality among those who were receiving either invasive mechanical ventilation (IMV) or oxygen alone at randomization, but not among those receiving no respiratory support [73]. After this publication, SSC guidelines on the management of COVID-19 in ICUs were updated and strongly recommended systemic corticosteroids [74]. There were, however, some doubts regarding the administration of systemic corticosteroids in this setting. A multicenter, single-blind, randomized control trial demonstrated that the early use of corticosteroids may prolong SARS-CoV-2 shedding in non-ICU patients with COVID-19 pneumonia [75]; in the methylprednisolone group, the median was 11 days, which was significantly longer than that of the control group (8 days). Viral RNA load in plasma is associated with critical illness and a dysregulated host response in COVID-19 [76]. The viral RNA load in plasma correlates with higher levels of chemokines CXCL10 and CCL2 (CXCL10 is interferon-γ-inducible protein 10, previously called IP-10; CC chemokine ligand 2—CCL2—is also known as monocyte chemotactic protein-1), as well as the biomarkers of a systemic inflammatory response (IL-6, CRP, ferritin). A significantly higher viral RNA load was present in ICU patients compared with ward and outpatients. The authors of that study proposed an integrative model of correlation between higher SARS-CoV-2 RNA plasma load on one hand and higher cytokine, CRP, ferritin, d-dimer, LDH (tissue damage) and endothelial dysfunction marker levels on the other hand. A higher viral load was also associated with activated neutrophil responses and lymphopenia. In a recently published study, it was demonstrated that a longer viremia duration was associated with mortality in hospitalized COVID-19 patients. The median time to serum viral clearance was 7 days after admission. The odds of mortality increased by 40% for each additional day of viremia [77]. In addition, fungal co-infections, such as invasive aspergillosis, mucormycosis, candidiasis or cryptococcosis, are associated with the COVID-19 pandemic [78,79]. These findings support the identification of the patients who should be treated with corticosteroids. In one study, the investigators identified hypoinflammatory and hyperinflammatory phenotypes in critically ill COVID-19 patients. They demonstrated that corticosteroid therapy was not associated with 28-day mortality in the overall population, but the administration of corticosteroids showed significant survival benefits only in patients with the hyperinflammatory phenotype (i.e., elevated cytokine, CRP and d-dimer levels) [80]. Evidently, in the severe COVID-19 setting, one size does not fit all; the answer is, as in other fields of medicine, moving toward a personalized approach for steroids in COVID-19 patients [81]. In another interesting study, a total of 2017 COVID-19 ICU patients were classified as A—severe phenotype, B—critical phenotype and C—life-threatening phenotype. The ICU crude mortality for the overall patient population was 32.6%: 20.3% for the A phenotype, 25.5% for the B phenotype and 45.4% for the C phenotype. The phenotype with the highest mortality was the hyperinflammatory one, with higher levels of CRP, d-dimer and ferritin. Furthermore, corticosteroids improved survival only in the C phenotype. The authors warned against the widespread use of corticosteroids in all critically ill patients with COVID-19 at moderate doses [82]. Having guidelines should not prevent us from exploring and critically analyzing the evidence further [83]. For instance, in the RECOVERY trial [73], regarding corticosteroids vs. SOC in severe COVID-19 patients, an imbalance in the non-reported/measured factors (body mass index (BMI), ethnic origin) may have also occurred. Given the fact that COVID-19 patients on invasive mechanical ventilation benefit the most from dexamethasone, it is interesting that key factors such as positive end-expiratory pressure (PEEP), oxygen inspiratory fraction (FiO_2_), partial pressure of oxygen in the arterial blood—PaO_2_/FiO_2_—pH, partial pressure of carbon dioxide in the arterial blood—PaCO_2_—plateau pressure, prone position, etc., are not reported. This might be important because the RECOVERY trial accounted for 57% of the results in the meta-analysis [84], in which it was demonstrated that the administration of systemic corticosteroids (dexamethasone, hydrocortisone, methylprednisolone, i.e., class effect), compared with SOC, was associated with lower 28-day all-cause mortality, so it is a major player with smaller satellites. A critical review of evidence regarding corticosteroids in severe COVID-19 was concisely performed by De Backer et al. [83]. The authors pointed out that the long-term impact of steroids in COVID-19 should be taken into account. Another important point they raised regards 28-day mortality being the best endpoint. Except for REMAP-CAP, all trials included in the meta-analysis focused on 28-day mortality or less. In the REMAP-CAP trial, 45% of the patients were still in the ICU on day-28. In general, mortality rates differ significantly between day-28 and hospital discharge. Thus, hospital mortality might be more valid in a critical care setting. Corticosteroids might have a short-term beneficial effect on ventilation but detrimental long-term effects and might fail to affect day-90 mortality. A recently published study focused on a growing number of COVID-19-associated pulmonary aspergillosis (CAPA) cases [85]. The authors clearly identified corticosteroids as a risk factor because they demonstrated that dexamethasone therapy, as recommended for COVID-19, was associated with a significant three-fold increase in the risk of CAPA. As to the question of whether we can identify which patients benefit most from corticosteroid therapy in COVID-19, a recent latent class analysis revealed COVID-19-related acute respiratory distress syndrome (CARDS) subgroups with differential responses to corticosteroids [86]. The full story about corticosteroids in viral and other forms of sepsis is far from over.

### 3.2. Intravenous Immunoglobulins (IVIGS) as Adjunctive Therapy

Viral infection in a COVID-19 setting is transformed into an immune pathology in a severe form, so there is a viral response phase as well as a host inflammatory phase. In severe-to-critical COVID-19, there is a cytokine breeze, not a storm as in polymicrobial sepsis; furthermore, there is a substantial anti-inflammatory pattern from the very beginning [87]. Immunoglobulins can attenuate as well as enhance the immune response. Intravenous immunoglobulins (IVIGS) in ICU COVID-19 might be an adjunctive therapy in later stages. One study investigated the early administration of high-dose IVIG in critically ill COVID-19 patients. The authors concluded that this therapeutic procedure improved the prognosis of these patients [88]. In another, similar study, the authors demonstrated that high-dose IVIGs administered in severe COVID-19 patients within 14 days of onset were linked to reduced 28-day mortality [89]. The rationale for IVIGs in COVID-19 was investigated in a meta-analysis that retrieved four clinical trials and three cohort studies including 825 hospitalized patients. The authors demonstrated that the severity of COVID-19 is associated with the efficiency of IVIG. In a critical subgroup analysis, IVIG was found to reduce mortality compared with a control group [90]. These were all retrospective studies, so it is important to emphasize that in one higher-quality, recently published, prospective, double-blind, placebo-controlled phase 3 trial, the authors demonstrated that in COVID-19 patients on invasive mechanical ventilation for moderate-to-severe ARDS, IVIGs (mainly IgG) did not improve clinical outcome at day-28 [91]. This negative result might be explained by the fact that only patients on mechanical ventilation were included, so at that phase, it might be too late for IVIGs. Furthermore, the beneficial effects of IVIGs might depend on the composition of the IVIG preparation [92]. In the COVID STEROID 2 randomized trial [93], the investigators found that among patients with COVID-19 and severe hypoxemia, 12 mg/d of dexamethasone compared with 6 mg/d of dexamethasone did not result, statistically, in significantly more days alive at 28-days. An interesting systemic review and meta-analysis regarding COVID-19 as well as non-COVID-19 ARDS and corticosteroids revealed that corticosteroids probably reduce mortality and the duration of mechanical ventilation in both COVID and non-COVID ARDS. Thus, these immunomodulating drugs should likely be used in most patients with ARDS, regardless of etiology [94].

### 3.3. Anti-IL-6 Therapy

Biological drugs against IL-6 can target the cytokine directly (siltuximab, clazakizumab, sirukumab and olokizumab) while membrane-bound IL-6 receptor is blocked by tocilizumab and sarilumab [47,95]. Soluble IL-6R is blocked by olamkicept, resulting in the arrest of IL-6 trans-signaling [95]. All of these drugs will, to some extent, increase IL-6 concentration in plasma. Given that peak IL-6 levels in the majority of COVID-19 patients are less than 100 pg/mL, it is a cytokine breeze or drizzle, not a storm. A key meta-analysis regarding biological drugs against IL-6 encompassed almost 11,000 hospitalized COVID-19 patients from 27 trials [96]. All-cause 28-day mortality was 21.8% in the IL-6 antagonists’ arms versus 25.8% in the SOC arms. The meta-analysis confirmed that a significant mortality benefit was only found when IL-6ra was co-administered with glucocorticoids and in patients with progressive COVID-19. Therefore, these drugs should not be given too early or too late (patients on prolonged MV). Thus, as with the other drugs, timing is everything [97].

### 3.4. Selective Janus Kinase (JAK) 1/2 Inhibitor—Baricitinib

The baricitinib story is quite interesting. Early in the pandemic, at the beginning of February 2020, investigators used artificial intelligence (AI) platforms to determine the likelihood of hundreds of different drugs to be repurposed for COVID-19 treatment. The BenevolentAI knowledge graph integrates biomedical data from structured and unstructured sources. Richardson et al. used the AI platform to find drugs with the potential to inhibit SARS-CoV-2 and reduce inflammation [98]. Baricitinib was identified as the best candidate from a long list of hundreds of potential medications. Subsequently, in vitro assays confirmed that baricitinib inhibits viral entry and impairs viral endocytosis and propagation. In addition, baricitinib is a selective Janus kinase (JAK) 1/2 inhibitor that prevents the activation of the signal transducer and transcription (STAT) pathway, which has systemic proinflammatory effects. Therefore, it has anti-inflammatory effects by inhibiting an array of cytokines involved in myeloid dysregulation, endothelial inflammation and antigen presentation. Key characteristics of this JAK inhibitor include oral tablet administration once a day, a short half-life of approximately 12 h, few drug-drug interactions and a well-established safety profile. In a retrospective, multicenter study, baricitinib reduced COVID-19 mortality rates, as well as ICU admissions in patients with COVID-19 pneumonia. [99]. COV-BARRIER was a randomized, double-blind, parallel-group, placebo-controlled phase 3 trial regarding the efficacy and safety of baricitinib for the treatment of hospitalized adults with COVID-19 [100]. The investigators recruited more than 1500 patients who were randomly assigned 4 mg of baricitinib orally administered for 14 days or a SOC group. Patients enrolled were hospitalized adults with SARS-CoV-2, evidence of pneumonia or active COVID infection and increased levels of inflammatory markers (CRP, d-dimer, LDH, ferritin). Patients not requiring supplemental oxygen were excluded. A sub-study of 101 patients on IMV or ECMO was added, and the results were recently published [101]. Increasing illness severity yielded an increased absolute risk reduction in death: there was a greater separation of groups with lower mortality in the baricitinib group. For hospitalized COVID-19 adults, the 28-day all-cause mortality was 8% for baricitinib versus 13% for the placebo; one additional death was prevented for 20 baricitinib-treated participants. More importantly, the 60-day, all-cause mortality remained lower for baricitinib (10%) versus the placebo (15%). In critically ill hospitalized patients with COVID-19 who were on IMV or ECMO, treatment with baricitinib, compared with the placebo (in combination with standard of care, including corticosteroids), also reduced mortality. Treatment with baricitinib significantly reduced 28-day, all-cause mortality compared with the placebo (29% vs. 39%) as well as 60-day mortality (45% vs. 62%). In every six baricitinib-treated participants, one additional death was prevented compared with the placebo at days 28 and 60 (NNT—number needed to treat to save a life).

### 3.5. Anti-IL-1 Therapy—Anakinra

Anakinra is a recombinant form of IL-1 receptor antagonist (IL-1ra) with a short half-life and a good safety profile [102]. It was proposed by experts to use anakinra in hospitalized COVID-19 patients before ICU admission. This immunomodulating drug has only mild immunosuppressive effects, so its ability to clear bacterial or fungal infections is not impaired. Unlike tocilizumab, anakinra has a short half-life and can be stopped fast; since IL-1 is a potent IL-6 inducer, anakinra will also decrease IL-6 production. The blocking actions of IL-1 by anakinra will result in preventing IL-1-inflammasome-mediated disease. Inflammasomes play a central role in the production of IL-1β because they activate caspase-1, which cleaves inactive pro-IL-1β to active IL-1β. During rapid SARS-CoV-2 replication, lung epithelial cells release DAMPs or alarmins, which act on Toll-like receptor (TLR)-4. This cascade results in the intracellular accumulation of pro- IL-1β [103]. Parallel to this cascade, under stimulation from DAMPs, IL-1α is produced. This cytokine is already active and does not need any further processing. The release of abundant quantities of IL-1α has been described in lung infections, including COVID-19 [104]. As early as spring 2020, several case reports regarding the use of anakinra in patients with severe COVID-19 were published. [105,106,107]. These reports demonstrated that anakinra led to an improvement in patients deemed to be in a hyperinflammatory state based on high ferritin and CRP values. It should be noted that both parameters of inflammation are routine and easily obtainable. One retrospective cohort study included COVID-19 patients treated with at least one dose of tocilizumab or anakinra for COVID-19-related so-called cytokine storm (COVID19-CS) [108]. After accounting for differences in disease severity at treatment initiation, the apparent superiority of anakinra over tocilizumab lost its statistical significance, but anakinra was not inferior to tocilizumab. Here, the authors also relied on routine biomarkers of inflammation (ferritin, CRP, d-dimer, neutrophil count, etc.). The authors of a systematic review and patient-level meta-analysis of the effect of anakinra on mortality in patients with COVID-19 [109] aggregated data on 1185 patients from nine studies [22,110,111,112,113,114,115,116,117]. They demonstrated that mortality in patients treated with anakinra was significantly lower in comparison to SOC and/or placebo groups at 11.1% versus 24.8%, respectively. Out of nine included studies, lower mortality rates in anakinra groups were found in seven. In one small-sample-sized study [110], there was no mortality in an anakinra group of 12 patients, while the SOC group consisted of 10 patients. The other remaining study was CORIMUNO-ANA-1 [22], a randomized control trial in which there was no statistically significant difference in mortality between groups (19% in the anakinra group vs. 18% in the control group), so this RCT was deemed negative. There are several possible explanations for these findings: no biomarker was used for the selection of patients, so it is possible that some of the enrolled patients were not at a stage of early IL-1 activation; there was also no placebo, and the anakinra treatment was short, only 5 days [118]. Significantly higher survival at day-28 [119] and clinical improvement [120] in anakinra-treated groups were demonstrated in two retrospective cohort studies of COVID-19 patients. In one study, the investigators compared an IL-1 inhibitor anakinra group with an IL-6 inhibitor tocilizumab or sarilumab group; the third was a SOC group of hospitalized patients with severe COVID-19. They demonstrated that IL-1 inhibition, but not IL-6 inhibition, was associated with a significant reduction in mortality [113]. In patients with tocilizumab-refractory severe COVID-19, anakinra as a salvage therapy failed to improve survival [121].

In order to predict the outcomes of patients with severe COVID-19, information regarding inflammation, lymphocyte function and coagulation needs to be collected. There is one biomarker that integrates all this information, the aforementioned suPAR. Rovina et al. collected serum samples from COVID-19 patients within the first 24 h of admission, and suPAR levels were measured [122]. At that time, no severe respiratory failure (SRF) was present (a PO_2_/FiO_2_ (P/F) ratio higher than 150 means there is no need for MV or continuous positive airway pressure treatment (CPAP)). The authors demonstrated that suPAR is an early predictor of the development of severe respiratory failure. Huang et al. highlighted the fact that suPAR DI-III is an active form of suPAR [123]. Another group of authors also emphasized the fact that the suPAR level increases with the severity of the infection or organ dysfunction, reflecting the body’s immune response, and may serve as an independent marker of clinical severity and outcomes in COVID-19 patients [124]. suPAR is an immune mediator of acute kidney injury (AKI). Given that almost half of hospitalized COVID-19 patients develop AKI, with 20% requiring dialysis, this biomarker can also be predictive of the need for dialysis [125]. Another validation of suPAR as a biomarker in this setting was published recently [126], focusing on patients with diabetes mellitus (DM) hospitalized for COVID-19. The authors concluded that the association between DM and outcomes in COVID-19 is largely mediated by hyperinflammation, as assessed by suPAR levels. Thus, in the pivotal SAVE-MORE RCT of anakinra in hospitalized COVID-19 patients, suPAR appeared to be the logical choice for a biomarker. The investigators conducted the suPAR-guided anakinra treatment and demonstrated that 28-day mortality was lower (3.2% in anakinra arms versus 6.9% in controls) [23]. Over 80% of patients in the placebo and anakinra arms also received standard-of-care glucocorticoids, yet anakinra still notably improved survival and shortened hospital stays [127]. Treatment with anakinra was hence associated with survival benefits in severe COVID-19. This suggests that the IL-1 pathway is already activated in severe forms of the disease and that the activation of IL-1 may start before signs of severity appear. Furthermore, the peak of IL-1β appears to precede the peak of other cytokines [118]. Interestingly, in the CAN-COVID trial [128], patients with hypoxic COVID-19, but who were not on IMV and who had hyperinflammation parameters present, were randomized to a placebo group or provided a single dose of the intravenous anti-IL-1β monoclonal antibody canakinumab, which showed no benefit. The authors concluded that treatment with canakinumab, compared with a placebo, did not significantly increase the likelihood of survival without IMV at day-29. Yet, it has to be noted that canakinumab inhibits only IL-1β, while anakinra exhibits activity against both IL-1α and IL-1β. In addition, the CAN-COVID trial was not guided by suPAR. Despite all the evidence regarding anakinra, the latest update of the COVID-19 Treatment Guidelines Panel (31 May 2022) did not change previous its guidance that there is insufficient evidence to recommend either for or against the use of anakinra for the treatment of COVID-19. As SAVE-MORE is a pivotal RCT for anakinra, the COVID-19 Treatment Guidelines Panel stated only the following limitation: “The laboratory assay that is used to assess suPAR levels is not currently available in many countries, including the United States”. Thus, it seems that the suPAR assay is the problem. The SAVE-MORE investigators offered a replacement for suPAR as the solution. They recently created the SCOPE (Severe Covid Predictor Estimate) Score [129]. Giamarellos-Bourboulis et al. introduced the SCOPE score (0–12 points) for the early prognostication of the risk for severe respiratory failure or death within the next 14 days in COVID-19 pneumonia. This is composed of CRP, d-dimer, ferritin and IL-6 concentrations. Anakinra should be administered when the SCOPE score is 6 points or more because then there is a six-fold risk of severe respiratory failure or death. This score can be used as an alternative to suPAR. Each of the four biomarkers is allocated 0 to 3 points according to the concentration. The final score is the sum of the points provided by each biomarker. For example: d-dimer >0.90 mg/L (3 points), CRP >85 mg/L (3 points), ferritin >750 ng/mL (3 points) and IL-6 >30 pg/mL (3 points) represent the worst-case scenario. A SCOPE score higher than 6 points and suPAR concentration higher than 6 ng/mL have the same, very good AUC/ROC value of 0.81. Fortunately, the human medicines committee of the European Medicines Agency (EMA) recommended extending the indication of anakinra to include the treatment of COVID-19 in adult patients on 17 December 2021. Kineret^®^ is now authorized across the European Union to treat COVID-19.

An individualized approach to each patient is paramount. An excellent example of an ongoing multicenter and multinational, double-blind, double-dummy randomized clinical trial on personalized immunotherapy in sepsis is the IMMUNOSEP trial [130]. Septic patients with either pneumonia or primary bloodstream infections are classified into either a macrophage-activation-like syndrome group or a sepsis-induced immunosuppression group. The idea is not to randomize patients according to drugs but according to strategy. Patients with macrophage-activation-like syndrome receive intravenous anakinra or placebos, and those with hypoinflammation receive subcutaneous recombinant interferon (IFN)-γ or placebos. So far, one out of four enrolled patients has been classified into the macrophage-activation-like syndrome group; therefore, the majority of patients are immunosuppressed.

### 3.6. Anticoagulant and Antiplatelet Agents

Anticoagulant and antiplatelet agents are an important part of COVID-19 treatment. Early information during the COVID-19 pandemic came from a retrospective study in Tongji Hospital, Wuhan [131], in which 449 patients with severe COVID-19 were enrolled. Only 22% of these patients received heparin, and it was associated with decreased mortality. Strong evidence regarding therapeutic anticoagulation with heparin in critically ill COVID-19 patients came from an open-label, adaptive, multiplatform, randomized clinical trial conducted by the REMAP-CAP, ACTIV-4a and ATTACC investigators [132]. The investigators demonstrated that there was no benefit to the initial strategy of therapeutic-dose anticoagulation with heparin (over 3% of patients in that arm experienced major bleeding) compared with SOC pharmacologic thromboprophylaxis. In noncritically ill COVID-19 patients (on the wards, with supplemental oxygen but without any form of mechanical support), however, an initial strategy of therapeutic-dose anticoagulation with heparin increased the probability of survival to hospital discharge with the reduced use of cardiovascular or respiratory organ support compared with SOC thromboprophylaxis [133]. The RAPID randomized clinical trial [134] also demonstrated that, in moderately ill COVID-19 patients admitted to hospital wards and with increased d-dimer levels, therapeutic heparin resulted in decreased odds of death at 28 days. The risk of major bleeding appeared low in this trial. The INSPIRATION trial focused on intermediate-dose vs. SOC prophylactic anticoagulation in ICU COVID-19 patients [135]. Intermediate-dose prophylactic anticoagulation did not perform better than SOC prophylactic anticoagulation, and hence, it should not be used routinely in unselected ICU patients with COVID-19. An interesting study on the direct oral anticoagulants (DOACs) rivaroxaban or enoxaparin in patients hospitalized with COVID-19 with elevated d-dimer concentrations was performed in Brazil [136]. The investigators demonstrated that in-hospital therapeutic anticoagulation with rivaroxaban or enoxaparin followed by rivaroxaban on day-30 did not improve clinical outcomes and increased bleeding compared with prophylactic anticoagulation; therefore, the use of a therapeutic dose of DOACs should be avoided in these patients. The intriguing fact that low-molecular-weight heparin (LMWH), but not DOACs, is beneficial in moderate COVID-19 cannot be explained by the antithrombotic effect. It is possibly the anti-inflammatory effect of LMWH that is important for the aforementioned immunothrombosis in COVID-19 [137]. As far as antiplatelet agents in the treatment of COVID-19 are concerned, there are two recently published relevant major trials. The first one focused on aspirin use in the RECOVERY trial [138]. There was no evidence that aspirin treatment reduced mortality at 28 days in hospitalized COVID-19 patients. The results were consistent in all pre-specified subgroups of patients. In fact, for every 1000 patients treated with aspirin, approximately 6 more patients experienced a major bleeding event, and approximately 6 fewer experienced a thromboembolic event. More recently published was the REMAP-CAP trial regarding the effect of antiplatelet therapy on survival and organ-support-free days in critically ill patients with COVID-19 [139]. Critically ill patients with COVID-19 who received anticoagulation therapy were randomly allocated to receive aspirin (at doses between 75 mg and 100 mg; *n* = 565), to receive 1 of 3 P2Y12 inhibitors (clopidogrel, 75 mg; ticagrelor, 60 mg; or prasugrel, 60 mg; *n* = 455), or to an open control group (*n* = 529). There was no benefit from any form of antiplatelet agent. Moreover, 2% of the patients receiving antiplatelet agents experienced major bleeding events versus 0.4 in the control arm. Hence, standard LMWH thromboprophylaxis should be used in critically ill patients with COVID-19, and full-dose LMWHs in moderate or ward COVID-19 patients as additional antiplatelet agents are of no benefit. One of the major revisions of guidelines issued by the COVID-19 Treatment Guidelines Panel on 31 May 2022 was the recommendation against the use of antiplatelet therapy to prevent COVID-19 progression or death in noncritically ill patients. There was insufficient evidence for the Panel to recommend either for or against antiplatelet therapy in critically ill patients with COVID-19 [71]. The pathophysiology of COVID-19-induced lung injury is quite specific. SARS-CoV-2 needs to enter the cell in order to replicate. By hijacking the ACE2 receptor of the alveolar cell, the virus immediately causes lung injury because angiotensin II cannot be broken down, and that causes vasoconstriction. Fibrin and hyaline membrane formation inside the alveolar sacs attract neutrophils, and this part of complex coagulopathy is much more pronounced in COVID-19 versus non-COVID-19 ARDS. This process is complicated by micro- and macrovascular thrombosis in the pulmonary vasculature. During the first year of the pandemic, it became clear that COVID-19 ARDS is characterized by significantly more coagulopathy, thrombosis and pulmonary embolisms versus non-COVID-19 ARDS [140]. After the activation of macrophages and after neutrophils enter alveoli, pulmonary cytokine overproduction is present but typically does not spill over into the systemic circulation. The formation of very toxic DNA NETs causes further lung injury. Potential therapeutics would hence include antivirals, anti-inflammatory drugs and anticoagulants. Heparin is unique in this regard, as it has all three effects as well as a mucolytic effect (breaks down mucus). In view of this, investigators have focused on nebulized unfractionated heparin (UFH) as a treatment for COVID-19 [141]. Heparin prevents SARS-CoV-2 from entering the cell because it binds to and destabilizes the SARS-CoV-2 Receptor Binding Domain (RBD). Furthermore, investigators have demonstrated that heparin directly inhibits the binding of RBD to the human ACE2 protein receptor in cell culture. In addition, the antiviral activity of UFH is 100-fold stronger than LMWH [142]. There are two forms of heparin in human tissues; some heparin is free, stored in the mas cell granules of the liver, gut and respiratory tract, but most is attached to all cell membranes and connective tissues as heparan sulfate. Heparan sulfate is one of the largest molecules, with many long tentacles, and it acts like a chemical “mop”, collecting and concentrating biological molecules (antigens and microbes, for instance) on the cell surface to facilitate binding with other cell membrane receptors. Heparan sulfate collects and presents SARS-CoV-2 to the ACE2 receptor. Thus, if a large amount of free heparin is administered, heparan sulfate actions will be stopped, as free heparin will bind to the viral spike protein. There are many bacteria and viruses, including SARS-Cov-2, that bind heparan sulfate to infect human cells. Therefore, inhaled, nebulized heparin prevents SARS-CoV-2 from binding to heparan sulfate and ACE-2. Accumulated pre-COVID clinical evidence on nebulized heparin demonstrated a trend toward a reduction in coagulation as well as dead space in ARDS patients. There was also an increase in MV-free days [141]. The first phase 3 trial (pre-COVID) regarding nebulized heparin for patients with or at risk of ARDS was published in 2021 [143]. The aim of this study was to determine if nebulized heparin, which targets fibrin deposition, would limit lung injury in these patients. There was less progression of lung injury in the heparin group compared to the placebo. However, the primary outcome, the physical function of survivors at day 60, demonstrated that nebulized heparin did not improve the self-reported performance of daily physical activities. The first case series of nebulized heparin for the treatment of 98 hospitalized COVID-19 patients was very recently published [144]. The investigators demonstrated that inhaled nebulized heparin was safe in this patient population, and there was no clinically relevant increase in activated partial thromboplastin time (APTT), which is important because half of the patients were on concomitant therapeutic anticoagulation. There was also an improvement in oxygenation after UFH. A meta-trial in hospitalized non-ICU COVID-19 patients, INHALE-HEP, is ongoing [145].

### 3.7. Restoring Immune Response with Immunomodulators in the Fight against COVID-19

In the presence of viral pathogens, self-defense mechanisms include interferons (IFNs), most notably, type-I IFN [146]. Coronavirus suppresses the type-I IFN response, which is detrimental in COVID-19 patients. Another key point in this setting is SARS-CoV-2-induced lymphopenia (due to T cell apoptosis, necrosis, pyroptosis, etc.) with T cell exhaustion [147]. Type-I IFNs α/β are considered broad-spectrum antivirals with a direct inhibitory effect on viral replication and a supportive effect in immune responses [148,149]. In one of the first uncontrolled, exploratory studies, investigators treated 77 hospitalized COVID-19 patients with nebulized IFN-α2b [150]. They concluded that this treatment significantly reduced the duration of a detectable virus, as well as the duration of elevated IL-6 and CRP serum levels. The efficacy and safety of IFN-β1a in the treatment of severe COVID-19 were investigated in a randomized clinical trial [151]. Forty-two patients in the IFN arm had lower 28-day overall mortality compared with thirty-nine patients in the control arm. Type-I IFN can over-activate the inflammatory response; less toxic IFN III can be used instead of IFN I, with a similar antiviral effect [152]. Pegylated (PEG) IFN-λ1a was investigated in patients with mild-to-moderate COVID-19 [153]. The authors demonstrated that early administration within the first 3 days neither shortened viral shedding nor the duration of symptoms in these patients. It is worth mentioning that the protocol for the randomized control INTERCOP trial (IFNβ-1a in COVID-19 patients) was published in 2020 [154]. Unfortunately, after enrolling 56 participants, the trial was terminated due to futility (ClinicalTrials.gov Identifier: NCT04449380).

Immune exhaustion recovery is very significant, and ways to boost immune response are a research focus of investigators and clinicians [155]. IFN-γ (type-II IFN) is essentially an immunostimulatory agent that is commercially available. A very interesting report on six non-immunocompromised COVID-19 patients who sustained recurrent ventilatory associated pneumonia (VAP) and had low HLA-DR expression in monocytes was published last year [156]. These immunosuppressed patients were treated with 100 µg of subcutaneous IFN-γ for 5 consecutive days in order to restore activated monocytes. This intervention led to a fast increase in the proportion of HLA-DR^high^ monocytes in all but one patient. In another case series, five critically ill COVID-19 renal transplant patients with persistent high SARS-CoV-2 viral RNA loads (for 50–60 days with live SARS-CoV-2 during ICU stay) and no respiratory improvement were treated with IFN-γ, 100 μg subcutaneously, three times a week. The SARS-CoV-2 load rapidly declined in all patients, and there was a positive-to-negative viral culture conversion. Importantly, there were no signs of hyperinflammation in these patients [157]. Two other compounds under investigation that might boost immune response are checkpoint inhibitors [21] and recombinant IL-7. A study demonstrated that IL-7 can safely be administered to critically ill COVID-19 patients without exacerbating inflammation or pulmonary injury. IL-7 was associated with lymphocytes returning to a reference level, appearing to reverse a pathologic hallmark of COVID-19 [158].

## 4. COVID-19 Pandemic Brought Basic and Therapy Trials into the Spotlight

Scientific research should always be scrutinized and examined in detail with special attention to “small print”, and caution must be used when results are interpreted. COVID-19 research during the pandemic has been very challenging, producing many underpowered or duplicated studies with inconclusive results [159,160,161,162].

Over the last decade, some new and more efficient trial designs have emerged: basket trials (one agent; many diseases), umbrella trials (many agents) and, maybe most importantly for COVID-19 research, adaptive platform trials (many different interventions in the same patient population). The REMAP-CAP platform investigators recruited over 11,000 COVID and non-COVID patients, studied 55 different interventions in 16 different domains and generated conclusions about 10 interventions. There are new ways to measure success in therapy trials as an alternative to 28-day mortality as an endpoint; 90-day mortality is much better. Organ-support-free day (ordinal) scales are used in some COVID-19 RCTs to detect an effect. Days alive and at home after set time-intervals (3 months or 6 months for instance) is a more patient-oriented endpoint. Both ordinal scales and days alive integrate morbidity and mortality.

Biomarker-guided immunotherapy research on sepsis and COVID-19 appears to bring the concept of “one size fits all” to “one size does not fit all”. A very good example is an observational study regarding IL-6 serum levels as predictors of mortality and response to tocilizumab in COVID-19 patients [163]. A benefit was demonstrated in patients with low IL-6 levels who were not treated with tocilizumab and those with high IL-6 levels who were treated with tocilizumab. Their survival rates were the best. On the other hand, patients with low IL-6 levels treated with tocilizumab and those with high IL-6 levels who were not treated with tocilizumab had the worst prognosis. The authors concluded that baseline IL-6 greater than 30 pg/mL predicts IMV requirements in patients with COVID-19 and contributes to establishing an adequate indication for tocilizumab administration. This shows that some therapeutic interventions might have worked in the past for adequately selected patient populations; unfortunately, many of these therapeutic interventions were taken off the market. A good example is drotrecogin alfa—activated Xigris^®^—which was withdrawn due to a lack of efficacy over a decade ago, but it might work in some subtypes of septic patients. Maybe, one day, future studies will be based on the “one size fits one” concept.

Research methods have improved greatly since the beginning of the pandemic. Yet, there are lingering problems with the existing research methods. Only companies and large organizations can currently afford to undertake RCTs. Adaptive trial designs may not be useful when outcomes take a long time to observe (e.g., long COVID), arms are dropped early due to possibly misleading signals (e.g., anticoagulation) or increased practical complexity eliminates theoretical efficiency gains (e.g., non-invasive MV—NIV— and IMV). Living systematic reviews are only as good as the data they include (observations, selective cohorts, biased, latent factors, etc.), and there is the possibility of cognitive bias that investigators are unaware of. Scientific rigor is of paramount importance even in a pandemic. Well-established journals were as exposed to the retraction of SARS-CoV-2 and COVID-19 papers as lower journal impact factors (JIFs). The authors of the retracted articles had a moderately high h-index. The authors of the very interesting analysis in [164] stated that publication during a pandemic seems fraught with risk. The Editor in Chief of JAMA, Howard Bauchner, with Deputy Editors, Robert Golub and Jody Zylke, published an editorial concern regarding the possible reporting of the same patients with COVID-19 in different reports [165]. That practice, without clear disclosure of duplicate reporting, may affect subsequent estimates and preclude valid meta-analyses. The authors of an interesting retrospective, single-center study regarding experimental and compassionate drug use during the first wave of the COVID-19 pandemic [69] demonstrated that the administration of multiple experimental/compassionate medications to the same patient not only does not improve survival rates but the true effect of any single drug remains questionable even after adjusting for the receipt of additional treatments. In these circumstances, it is more difficult to identify a potential cure. In another JAMA editorial, it was pointed out that there may be significant heterogeneity in treatment effects based on the timing or constellation of disease manifestations [166]. Therefore, detailed patient characteristics are a very important part of reported trials because there might be patient subpopulations that might benefit more from therapeutic intervention. 

Baricitinib, remdesivir and anakinra have proven their efficacy in positive, placebo-controlled, double-blind randomized trials; corticosteroids and IL-6 have demonstrated probable efficacy in positive, open-label randomized trials. There are drugs with proven inefficacy in negative double-blind trials and in negative open-label trials: hydroxychloroquine (increased mortality up to 30%), azithromycin, ivermectin, convalescent plasma, interferon-beta-1a and therapeutic anticoagulation in critically ill patients. In an interesting editorial regarding the early adoption of critical care interventions [167], it was highlighted that it is unjustifiable to adopt these interventions without a concomitant effectiveness study. The author also pointed out that critical care clinicians are, in some ways, as diverse as the critical care patients for whom care is provided. There should be a concerted effort to de-adopt low-value strategies, but this is more difficult than it seems. Trials and endpoints are complex, so the results from a single trial need to be taken with a pinch of salt, and replicability in other trials is important.

During the COVID-19 pandemic, it became obvious that adaptive platform trials can collaborate with each other, and the concept of multiplatform RCTs emerged. Another element of the extraordinary global collaboration that occurred during the pandemic is the concept of the prospective meta-analysis (PMA). When multiple investigators are performing trials using the same agent and are willing to share their data in advance of publication, a PMA can be performed with published and unpublished trials.

More detailed examinations the of data in any meta-analysis are of paramount importance for drawing the correct conclusions from all aspects. For example, a meta-analysis on systemic corticosteroids and mortality in critically ill COVID-19 patients [84] showed that fungal infections were evaluated in only three out of seven RCTs considered, and viral reactivation was not evaluated at all in any of the seven RCTs. The same is true of a meta-analysis on IL-6 antagonists and mortality in hospitalized COVID-19 patients [96]; fungal infections were evaluated in only 5 out of 22 RCTs considered for infections, and viral reactivation was evaluated in only 1 of these 22 RCTs (20 patients). The authors of a recently published review on pharmacological studies in hospitalized COVID-19 patients in Belgium emphasized that more efforts could have been made to avoid running small, underpowered, mono- or bicenter trials to create better national collaboration and to participate in more international clinical trials and, more specifically, in adaptive, platform trials [168].

## 5. Discussion

A very interesting viewpoint by Andre Kalil regarding different therapeutic attempts was published at the beginning of the COVID-19 pandemic [169]. He strongly opposed a common interpretation of off-label use and the compassionate use of drugs; i.e., if the patients died, they died from the disease, but if the patients survived, they survived because of the given drug. He is also convinced that open-labeled, randomized trials inherently suffer from unconscious human bias when investigators know what the patients are receiving. Mervyn Singer and Andre Kalil wrote an excellent editorial pointing out that a plethora of interventions, many with next-to-no scientific basis or even directly conflicting effects have been trialed or, worse still, simply given to the patients on compassionate grounds [170]. It became clear to clinicians and researchers that COVID-19 is not a linear disease, and to view it as such is an oversimplification. This infection follows non-linear dynamics as immune response may be dysregulated early and viral load may be high late. The SARS-CoV-2 viral load in the upper respiratory tract appears to peak in the first week of illness, with a very long mean duration of SARS-CoV-2 RNA shedding over 17 days [171]. There are different viral loads in different compartments of the body. One of the important issues is how and when to combine therapies for COVID-19. When baricitinib was added to the antiviral remdesivir for hospitalized COVID-19 patients, a double-blind RCT demonstrated that baricitinib plus remdesivir was superior to remdesivir alone [172]. Interestingly, when the same was done with tocilizumab, an RCT demonstrated that adding tocilizumab to remdesivir did not produce any extra benefits compared to remdesivir alone [173]. In both the RECOVERY [174] and REMAP-CAP [175] trials, patients who received both tocilizumab and corticosteroids had lower mortality. Data examining those who received only tocilizumab are more consistent with harm, so an interaction between corticosteroids and tocilizumab exists. In other words, tocilizumab probably does not work without corticosteroids. On the other hand, there is no interaction between baricitinib and corticosteroids; therefore, in the COV-BARRIER trial [100], it was demonstrated that baricitinib reduced mortality with or without baseline corticosteroid use. Most recently (3 March 2022), an RCT regarding baricitinib in patients admitted to hospital with COVID-19 (RECOVERY platform) was posted on medRixv [176]. The authors demonstrated that, in patients hospitalized for COVID-19, baricitinib significantly reduced the risk of death, but the size of the benefit was somewhat smaller than suggested by previous trials. Baricitinib shows significant additional benefits when added to remdesivir and when added to corticosteroids. On the contrary, tocilizumab does not show benefits when added to remdesivir or in the absence of corticosteroids. The advantages of anakinra have been highlighted throughout this review. García-García et al. compared two different strategies, anakinra vs. baricitinib, in the treatment of hospitalized COVID-19 patients [177]. The investigators observed similar mortality in all patients, i.e., anakinra and baricitinib demonstrated similar efficacy.

Finally, one very interesting and important aspect of interpreting the results of RCTs is the fragility index. It outlines the minimum number of participants in a positive clinical trial who would need to have had a different outcome for the results of the trial to lose statistical significance and is, therefore, helpful in interpreting the robustness of results [178]. A lower number on the fragility index indicates that the statistical significance of the trial depends on fewer events. For example, a score of 2 on this measure means that if two participants in the intervention group have different event outcomes, the RCT would not have a statistically significant result when using the conventional *p*-value cutoff of less than 0.05. In a recently published analysis on the fragility of the statistically significant results in COVID-19 RCTs [179], Itaya et al. performed a cross-sectional study of 47 RCTs with a total of 138,235 participants that had statistically significant results. The authors found that the median fragility index was 4; i.e., a median of four events was required to change the analysis findings from statistically significant to not significant. They concluded that these findings suggest that healthcare professionals and policymakers should not rely heavily on the individual results of RCTs for COVID-19.

## 6. Conclusions

Immunothrombosis is of paramount importance in COVID-19 because it is an effector pathway of the immune response to SARS-CoV-2. An interesting immunological time course analysis demonstrated overlapping but disparate inflammatory and immunosuppressive responses to SARS-CoV-2 and bacterial sepsis [180]. This is a very good example of why a personalized approach, the Holy Grail of modern medicine, is needed. During the course of COVID-19, the immune response changes from the early phase to the late phase in individual patients, so immunomodulating therapy should be guided by individual responses and might be different at different time points (early phase vs. late phase, for instance). Another important issue is the aging immune system and its ability to respond to SARS-CoV-2 infection. Of special interest is an overlap between severe COVID-19 and immunosenescence to highlight the risk of complications and death in older populations with COVID-19 [181]. From that perspective, the routine use of corticosteroid treatment in elderly COVID-19 patients is questionable. The COVIP study demonstrated higher 30-day mortality in critically ill elderly COVID-19 patients (aged 70 years or older) who received steroids as part of their treatment [182].

There are no simple answers to complex questions. Repurposing old drugs has been a very helpful approach in the COVID-19 pandemic. Immune responses in critically ill patients have been the focus of interest for researchers and clinicians alike [183], and this aspect took the center stage during the COVID-19 pandemic. EMA’s human medicines committee recommended extending the indication of anakinra to include the treatment of COVID-19 in adult patients. It is obvious that this is still a work in progress, with ongoing clinical trials. With over 6 million deaths from COVID-19, this is the right time to speed up this process.

## Data Availability

Not applicable.
